# Socioeconomic differences in the motivation to stop using e-cigarettes and attempts to do so

**DOI:** 10.1016/j.abrep.2020.100247

**Published:** 2020-01-12

**Authors:** Tina Jahnel, Stuart G. Ferguson, Timea Partos, Leonie S. Brose

**Affiliations:** a College of Health and Medicine, University of Tasmania, Hobart, Australia; b Addictions, Institute of Psychiatry & Neuroscience, King’s College London, London, UK

**Keywords:** Electronic cigarettes, Socioeconomic status, Motivation to stop, Attempts to stop

## Abstract

**Introduction:**

In England, the use of electronic cigarettes as a smoking cessation aid has become more popular than any other aid. Previous research suggests that ex-smokers from lower social groups are more likely to use e-cigarettes compared to ex-smokers from more socially advantaged groups. The present study aimed to assess the association between baseline education, income and employment status and (1) baseline motivation to stop using e-cigarettes (2) attempts to stop using e-cigarettes during follow-up among current smokers, recent ex-smokers and long-term ex-smokers who use e-cigarettes.

**Methods:**

UK online longitudinal survey of smokers, ex-smokers and e-cigarette users, May/June 2016 (baseline) and September 2017 (follow-up). In logistic regression models, motivation to stop using e-cigarettes at baseline (n = 994) and attempts to stop using e-cigarettes at follow-up (n = 416) among current smokers and ex-smokers were regressed onto baseline educational attainment, income, employment status while adjusting for baseline demographics, vaping status, smoking and e-cigarette dependence.

**Results:**

(1) Respondents with higher education (OR = 1.36; 95% CI: 1.06–1.74) or higher income (OR = 1.52; 95% CI: 1.17–1.98) were more likely to be motivated to stop using e-cigarettes, but only in unadjusted analysis. (2) Again, in unadjusted analysis only, employment was associated with reduced odds of attempting to stop using e-cigarette (OR = 0.50; 95% CI: 0.32–0.79).

**Conclusion:**

Higher socio-economic status may be associated with higher motivation to stop vaping but with lower likelihood of trying to do so.

## Highlights

• SES and motivation to stop using e-cigarettes may be associated.
• SES may predict attempts to stop using e-cigarettes.
• Further research on the social patterning of quitting vaping is needed.

## 1 Introduction

Despite public health efforts that have produced dramatic declines in the prevalence of cigarette smoking, it remains one of the leading causes of preventable disease and death in the developed world (GBD Tobacco Collaborators, 2017, Ng et al., 2014). Moreover, smoking is unequally distributed between people of different socioeconomic status with higher smoking prevalence in groups with lower socioeconomic status (SES); this contributes to health inequalities (Hiscock et al., 2012, Hiscock et al., 2015).

In England, the use of electronic cigarettes (e-cigarettes) as a smoking cessation aid has become more popular than any other aid, including nicotine replacement therapy (NRT) (McNeill, Brose, Calder, Bauld, & Robson, 2018). Daily use has been associated with increases in attempts to quit smoking and reducing smoking (Brose, Hitchman, Brown, West, & McNeill, 2015). Consistent with the diffusion of innovation model (Rogers, 2003) early studies found evidence of a social gradient in the use of e-cigarettes when they were first introduced (Adkison et al., 2013, Brown et al., 2014). These studies found that more socially advantaged “early adopters” of e-cigarettes showed greater awareness and use of e-cigarettes during a quit attempt compared to less socially advantaged groups. However, this social gradient seems to have attenuated over time and today is no longer evident (Kock, Shahab, West, & Brown, 2018); that is, less socially advantage groups are now just as likely to use e-cigarettes as their more socially advantaged counterparts.

However, the same study found a social gradient in e-cigarette use among long term ex-smokers (Kock et al., 2019). Long-term ex-smokers from lower social groups were more likely to use e-cigarettes compared to ex-smokers from more socially advantaged groups. Kock et al. (2019) offered a possible explanation in which more affluent long-term ex-smokers might use e-cigarettes during their smoking cessation attempt and discontinue their use after successful smoking cessation, whereas less advantaged groups continue the use of e-cigarettes after successful smoking cessation. This would be consistent with the literature on smoking cessation which shows that smokers from lower socioeconomic groups are less likely to successfully quit smoking than their more advantaged counterparts (Hiscock et al., 2015). To date, little evidence is available to evaluate whether a similar social gradient exists with the discontinuation of e-cigarette use. It is important to assess the social patterning in the discontinuation of e-cigarettes after a successful smoking cessation attempt, because although e-cigarettes are less harmful than smoking (Goniewicz et al., 2014) their use is not without risk (Farsalinos, 2018, Stephens, 2018). Thus, all else being equal, complete abstinence from not only smoking but also vaping would be most beneficial to health (Stephens, 2018) and could potentially have a positive effect on health inequalities (McNeill et al., 2018).

The aims of this exploratory study were to:

1. Assess the association between socioeconomic characteristics and motivation to stop using e-cigarettes among current smokers, recent ex-smokers and long-term ex-smokers who use e-cigarettes daily or non-daily.
2. Assess the association between socioeconomic characteristics and motivation to stop using e-cigarettes and subsequent attempts to stop using e-cigarettes among smokers, recent ex-smokers and long-term ex-smokers who use e-cigarettes daily or non-daily.

## 2 Methods

### 2.1 Design and sample

We conducted secondary analysis using data from an online longitudinal survey which recruited from a panel managed by Ipsos MORI (Brose et al., 2017, Lee et al., 2019). For the larger study all members who responded to an invitation to participate in a survey were screened and past-year smokers were eligible to participate. The invitation did not specify the topic of the survey. For Wave 4 (conducted in May/June 2016) and Wave 5 (conducted September 2017) current e-cigarette users who had never smoked were also eligible to participate. To be included in the current study, participants had to be either current smokers or ex-smokers (defined as having quit smoking either less than or more than three months ago) and current e-cigarette users (defined as using e-cigarettes daily or at least once a week). By completing the survey respondents would earn points which could be redeemed against high street gift vouchers or used to enter a prize draw. Quotas on age, gender, and UK region were imposed at recruitment stage in order to ensure representativeness. Data quality was ensured through standard protocols that included quality control checks, such as removal of responses where basics such as age did not match participants’ records or responses where the survey was completed in an unfeasible short amount of time.

Between November and December 2012, 23,785 respondents were invited of which 6165 past year-smokers were eligible to participate. Of the 5000 respondents who completed wave 1 of the survey, 2182 respondents completed the wave 2 in December 2013. The following year in December 2014, 1519 respondents were followed-up for wave 3. In May/June 2016 the sample was replenished for wave 4 resulting in a total of 3334 respondents completing the survey (27.9% re-contacted from wave 1 and 72.1% newly recruited). In September 2017, 1720 respondents were followed up for wave 5. The current paper analyses data from wave 4 (baseline data for this study) and wave 5 (follow-up).

Of the 3334 respondents at baseline, 3213 participants were categorized as either current smokers (n = 2429), long-term ex-smokers (defined as ex-smokers who had quit more than three months ago; n = 672) or recent ex-smokers (defined as ex-smokers who had quit within the last three months; n = 11 2). We used three months to distinguish between recent and long-term ex-smokers in order to allow for roughly even group sizes (Information Services Division, 2019). Although the cut-off points for long-term ex-smokers varies across the literature, previous research suggest that the majority of relapse occurs within the first 3 months following a quit attempt, with relapse after this period being much rarer (Jackson et al., 2019, Stapleton, 1998)*.* These findings suggest that after three months, the acute part of the quit attempt has been overcome, and people appear to be in a relatively stable non-smoking situation. Further, 649 current smokers, 291 long-term ex-smokers and 54 recent ex-smokers also used e-cigarettes daily or non-daily, resulting in a total sample of n = 994 respondents at baseline who were included in analysis to address aim 1. Of those, 493 participants were followed up. Due to inconsistent answers a total of n = 77 participants were excluded from the analysis for aim 2 (n = 26 respondents reported to use e-cigarettes daily or non-daily at baseline and reported to have never used e-cigarettes at follow-up; n = 51 respondents reported to use e-cigarettes daily or non-daily at baseline and reported to have only used e-cigarettes a few times at follow-up). This resulted in a follow-up sample of n = 416 respondents included in analysis for aim 2.

Ethics approval for the survey was obtained from King’s College London PNM Research Ethics panel. For waves 4 and 5 the codes are LRS15/162519 and LRS-16/17-4564.

### 2.2 Measures

The socioeconomic characteristics were measured at baseline and follow-up (Box S1). Baseline socioeconomic characteristics were used for analysis. Respondents provided their highest level of formal education, annual household income and employment status in the last 12 months. Multiple socioeconomic characteristics were used because they represent various facets of someone’s socioeconomic status which may have different implications on health and health behaviours (Duncan, Daly, McDonough, & Williams, 2002). Thus, the mechanisms that underly socioeconomic status and e-cigarette use may differ depending on which socioeconomic characteristics is used. Firstly, education may reflect the knowledge and skills a person has obtained, thereby affecting someone’s cognitive functioning, making them more receptive to health education messages and enabling the individual to communicate with or access health services (Galobardes, Shaw, Lawlor, Lynch, & Davey Smith, 2006). For example, education may determine a person’s health literacy; the ability to obtain, understand and use health information to make decisions relevant to stop using e-cigarettes (Nutbeam, 2000). Income on the other hand is a direct measure of access to scarce material resources such as health enhancing commodities and services. Income may influence health behaviours in various ways: Financial deprivation for example may motivate those individuals with few financial resources to either stop the use of e-cigarettes and thus safe resources or continue the use of e-cigarettes as a more affordable alternative to smoking cigarettes. Similarly, paid employment, highly correlated with income is a measure of financial resources and may determine someone’s ability to purchase e-cigarettes.

For the analysis highest educational attainment was collapsed into those with any university education and those without university education (including “Don’t know” and “Prefer not to say”). Including response options of ‘don’t know’ and ‘prefer not to answer’ as less than university education was based on a possible response bias for which we assumed that participants with very low education might feel uncomfortable disclosing their level of highest educational attainment. Income was collapsed into three categories based on the UK median disposable income in 2016 (Webber & Thomas, 2017). Employment status within the last 12 months prior to the baseline survey was dichotomized as “Yes” and “No” (including “Don’t know”; coded as 0).

To identify the three subgroups smokers, long-term ex-smokers and recent ex-smokers at baseline, participants were asked to indicate their current smoking status (Box S1). Current e-cigarette use was assessed at baseline and follow-up. Only baseline daily and non-daily e-cigarette users were included in the analysis. Non-Daily e-cigarette users had to report using e-cigarettes at least once a week in order to be considered a “current e-cigarette user”. Participants who reported using e-cigarettes once in the past month were not included in our sample. Motivation and intention to stop using e-cigarettes was measured at baseline using an adapted version of the Motivation and intention to stop scale (Kotz, Brown, & West, 2013) and dichotomized into “High motivation and intention to stop within the next 3 months (MITSE”) and “Low MITSE”. Attempts to stop using e-cigarettes was derived from two questions: Firstly, participants were asked about their vaping status at follow-up and those who indicated that they had stopped using e-cigarettes within the last year (“I have stopped vaping/using e-cigarettes in the last year”) were coded as having made an attempt to stop using e-cigarettes. Secondly, participants were asked about the number of serious attempts undertaken and were categorized as having made at none or least one attempt to stop between baseline and follow-up (Box S1).

Covariates included age (“18–24”; “25–39”; “40–54”, “55+”), gender (male, female), ethnic group (“White”; “Non-White”; “Don’t know/Prefer not to say”) and urges to use e-cigarettes/cigarettes. All smokers and ex-smokers who had quit less than one year ago were asked about their strength of urges to smoke (SUTS), which has been found to be a strong predictor of successful smoking cessation in population samples (Fidler, Shahab, & West, 2011). In addition, this scale is not dependent on number of cigarettes smoked per day which may be lower among smokers who also use e-cigarettes (Brose et al., 2015). E-cigarette dependence was measured using an adapted version of the SUTS questionnaire (Box S1).

### 2.3 Analytical plan

An attrition analysis was conducted to examine differences at baseline between respondents who were successfully followed-up versus lost to attrition using Pearson chi-square analyses (Table S1 in the supplementary material). Differences between current smokers, long-term ex-smokers and recent ex-smokers in baseline socioeconomic indicators and characteristics relating to e-cigarette usage were examined using Pearson chi-square analysis. To examine whether the smoker categories differed in terms of ethnicity, participants who responded to the ethnicity question: “Don’t know/Prefer not to say” (n = 14) were excluded. Additional sensitivity analysis was conducted with response options of ‘don’t know’ and ‘prefer not to answer’ for educational attainment recategorized as (a) having more than university education and (b) excluded from analyses.

Due to multicollinearity among the socioeconomic indicators, only one indicator at a time was entered in the multivariable logistic regression for Aim 1 and Aim 2.

*Aim 1:* To test whether indicators of socioeconomic status (education, income and employment status), among daily and non-daily e-cigarette users were associated with motivation and intention to stop using e-cigarettes at baseline, bivariate and multivariable logistic regression analyses were performed with age, gender, smoking status and urges to use e-cigarettes assessed at baseline included as covariates.

*Aim 2:* To test whether indicators of socioeconomic status (education, income and employment status), among daily and non-daily e-cigarette users were associated with attempts made to stop using e-cigarettes during follow-up, bivariate and multivariable logistic regression analyses were performed with age, gender, smoking status, motivation and intention to stop using e-cigarettes and urges to use e-cigarettes assessed at baseline included as covariates. Based on the results of the attrition analysis the results from the first aim, we conducted an interaction analysis in a separate model for Aim 2 in order to investigate potential combined effects of paid employment and motivation to stop using e-cigarettes. SPSS version 24 (IBM Corp, New York) was used for analyses.

## 3 Results

### 3.1 Sample and attrition

Compared with respondents who were lost to follow up between baseline and follow-up, respondents who were followed up were less educated, had less income, were more likely to have had paid employment in the last 12 months, were older, were more likely to be male and white (Table S1). There were no differences in terms of smoking status, vaping status, MITSE, urges to smoke or urges to use e-cigarettes.

In the baseline sample, 53.4% of respondents had a low level of educational attainment. Most participants were white males, had an income of £30,001 and over and had paid employment within the past 12 months (Table 1). Of the sample, 59% were using e-cigarettes daily, and nearly two thirds reported slight to moderate urges to use e-cigarettes. Half of the participants in the sample were motivated to stop using e-cigarettes. There were significant differences between smokers, recent and long-term ex-smokers in regard to educational attainment, age, ethnic group, vaping status, motivation and intention to stop using e-cigarettes and strength of urges to smoke. There were no significant differences between smoker type and income, paid employment within the last 12 months, gender and strength of urges to use e-cigarettes (Table 1). The sensitivity analyses where education was recategorized with response options of ‘don’t know’ and ‘prefer not to answer’ included as (a) having more than university education and (b) excluded from analyses did not significantly change the results for both aims. As such, we only report the findings with response options of ‘don’t know’ and ‘prefer not to answer’ included as low education.

**Table 1:** Characteristics of smokers, long-term ex-smokers and recent ex-smokers at baseline (n = 994).

		Total (N = 994) n (%)	Smokers (N = 649) n (%)	Long-term ex-smokers^a^ (N = 291) n (%)	Recent ex-smokers^b^ (N = 54) n (%)	Comparison
Education	Low (no University)	531 (53.4)	324 (49.9)	176 (60.5)	31 (57.4)	**χ2 = 9.366, *p* = .009**
	High (any University)	463 (46.6)	325 (51.1)	115 (39.5)	23 (42.6)	
Annual Income	Under £6501–30,000	443 (44.6)	280 (43.1)	142 (48.8)	21 (38.9)	χ2 = 3.345, *p* = .188
	£30,001 and over^c^	551 (55.4)	369 (56.9)	149 (51.2)	33 (61.1)	
Paid employment last 12 months	Yes	289 (29.1)	178 (27.4)	93 (32.0)	18 (33.3)	χ2 = 2.503, *p* = .286
	No	705 (70.9)	471 (72.6)	198 (68.0)	36 (66.7)	
Age	18–39	425 (42.8)	320 (49.3)	85 (29.2)	20 (37.0)	**χ2 = 33.918, *p* < .001**
	40–55+	569 (57.2)	329 (50.7)	206 (70.8)	34 (63.0)	
Gender	Female	416 (41.9)	259 (39.9)	136 (46.7)	21 (38.9)	χ2 = 4.055, *p* = .132
	Male	578 (58.1)	390 (60.1)	155 (53.3)	33 (61.1)	
Ethnic group^d^	White	896 (91.4)	574 (89.8)	264 (95.8)	46 (86.8)	**χ2 = 10.673, *p* = .005**
	Not White	84 (8.6)	65 (10.2)	12 (4.2)	7 (13.2)	
Vaping status	Daily	590 (59.4)	293 (45.1)	256 (88.0)	41 (75.9)	**χ2 = 159.244, *p* < .001**
	Non-daily	404 (40.6)	356 (54.9)	35 (12.0)	13 (24.1)	
MITSE	Want and intent to stop	492 (49.5)	333 (51.3)	122 (41.9)	37 (68.5)	**χ2 = 15.345, *p* < .001**
	Don’t want/intent to stop	502 (50.5)	316 (48.7)	169 (58.1)	17 (31.5)	
SUTS-E	Slight/Moderate^e^	715 (71.9)	455 (70.1)	222 (76.3)	38 (70.4)	χ2 = 3.870, *p****=***.144
	Strong/very strong/extremely strong	279 (28.1)	194 (29.9)	69 (23.7)	16 (29.6)	
SUTS^f^	Slight/Moderate^e^	517 (63.4)	366 (56.4)	104 (92.0)	47 (87.0)	**χ2 = 66.627, *p* < .001**
	Strong/very strong/extremely strong	299 (36.6)	283 (43.6)	9 (8.0)	7 (13.0)	

*Notes.* Significant associations (p < .05) are highlighted in bold. MITSE = Motivation and intention to stop using e-cigarettes within the next three months. SUTS-E = Strength of Urges to use e-cigarettes. SUTS = Strength of urges to smoke.

a Participants who stopped smoking completely more than three months before the last survey.

b Participants who stopped smoking completely three months or less before the last survey.

c Including “Don’t know/Prefer not to say.

d Participants who responded: “Don’t know/Prefer not to say” (n = 14) were excluded.

e Including No urges at all/Don’t know.

f Ex-smokers who stopped more than 1 year ago (n = 178) were not asked for urges to smoke.

### 3.2 Aim 1: Socioeconomic indicators and motivation to stop using e-cigarettes

Bivariate logistic regression suggested greater odds of being motivated to stop using e-cigarettes for respondents with higher education and higher income but not for those with paid employment (Table 2). However, in the multivariable logistic regression models, none of the socioeconomic characteristics were associated with motivation to stop using e-cigarettes. In the adjusted models, participants who were 25–39 years old had greater odds of being motivated to stop using e-cigarettes compared to 18–24-year-old participants, whereas older participants (55plus years) were less likely to want to stop using e-cigarettes in the unadjusted and adjusted models. Whilst only in the unadjusted model long-term ex-smokers were more likely to be motivated to stop using e-cigarettes compared to smokers, recent ex-smokers were more likely to be motivated to stop using e-cigarettes compared to smokers in the unadjusted and adjusted models. In all models, participants with strong urges to use e-cigarettes were more likely to be motivated to stop using e-cigarettes, whereas respondents with no urges (including “don’t know”) had smaller odds of being motivated to stop using e-cigarettes (Table 2).

**Table 2:** Baseline associations with motivation to stop using e-cigarettes (n = 994).

		Bivariate		Multivariate					
	Want to stop (%)	OR	95% CI	OR	95% CI	OR	95% CI	OR	95%CI
**Education (ref: Low)**									
High	53.6	1.36*	**1.06**–**1.74**	1.10	0.84–1.44				
**Income (ref: Low)**									
High	55.4	1.52**	**1.17**–**1.98**			1.21	0.91–1.61		
Don’t know/Prefer not to say	39.7	0.81	0.49–1.32			0.78	0.47–1.31		
**Paid employment last 12 months (ref: No)**									
Yes	39.8	0.58***	**0.44**–**0.76**					0.78	0.57–1.08
**Age (ref: 18–24)**									
25–39	63.6	1.52	0.97–2.39	1.64*	**1.02**–**2.63**	1.62*	**1.01**–**2.60**	1.64*	**1.02**–**2.63**
40–54	45.2	0.72	0.46–1.12	0.78	0.49–1.24	0.77	0.48–1.23	0.78	0.49–1.24
55plus	34.5	0.46***	**0.29**–**0.74**	0.53*	**0.32**–**0.86**	0.54*	**0.33**–**0.88**	0.58*	**0.35**–**0.96**
**Gender (ref: Male)**									
Female	46.4	0.81	0.63–1.04	0.92	0.70–1.20	0.93	0.71–1.22	0.93	0.71–1.22
**Smoking status (ref: Smoker)**									
Long-term ex-smoker	41.9	0.69**	**0.52**–**0.91**	0.77	0.55–1.06	0.77	0.56–1.07	0.76	0.55–1.05
Recent ex-smoker	68.5	2.07*	**1.14**–**3.74**	2.02*	**1.09**–**3.78**	1.99*	**1.07**–**3.71**	2.04*	**1.09**–**3.81**
**Vape Status (ref: Daily)**									
Non-Daily	49.3	0.98	0.76–1.27	1.11	0.82–1.51	1.11	0.82–1.52	1.11	0.82–1.51
**Strength of urges to use e-cigarettes (ref: Slight/Moderate)**									
No urges at all/Don’t know	21.6	0.28***	**0.17**–**0.45**	0.24***	**0.15**–**0.39**	0.24***	**0.15**–**0.39**	0.24***	**0.15**–**0.39**
Strong	61.8	1.65*	**1.19**–**2.28**	1.48*	**1.06**–**2.08**	1.46*	**1.04**–**2.05**	1.48*	**1.06**–**2.08**
Very/extremely strong	58.3	1.42	0.87–2.34	1.29	0.77–2.17	1.30	0.77–2.19	1.30	0.77–2.20

*Notes.* Ref = reference group. OR = odds ratio. LCI = Lower confidence interval. UCI = Upper confidence interval. Significant associations (p < .05) are highlighted in bold. Multivariable Models were run with one socioeconomic indicator at a time. * *p* < .05, ** *p* < .01, *** *p* < .001.

### 3.3 Aim 2: Socioeconomic indicators and attempts to stop using e-cigarettes

In the bivariate model, neither educational attainment nor income at baseline was a significant predictor of attempts to stop using e-cigarettes at follow-up. In the unadjusted model, compared to respondents who did not have paid employment in the 12 months leading up to the baseline survey, respondents with paid employment had lower odds to undertake an attempt to stop using e-cigarettes (Table 3). The findings from the three multivariable models for each socioeconomic indicator mainly confirm the findings from the bivariate logistic regressions. However, when adjusting for all covariates, having paid employment within the 12 months before baseline was no longer associated with an attempt to stop using e-cigarettes during follow-up. Further, the interaction analysis showed no significant association between socioeconomic indicators and attempts to stop using e-cigarettes. In all three multivariable models, age was no longer significantly associated with attempts to stop using e-cigarettes.

**Table 3:** Baseline associations with attempts to stop using e-cigarettes at follow up (n = 416).

		Bivariate		Multivariate					
	At least one attempt to stop (%)	OR	95% CI	OR	95%CI	OR	95%CI	OR	95% CI
**Education (ref: Low)**									
High	38.0	1.25	0.84–1.87	1.01	0.63–1.60				
**Income (ref: Low)**									
High	36.7	1.27	0.83–1.94			0.84	0.51–1.39		
Don’t know/Prefer not to say	47.5	1.98	0.99–3.96			2.02	0.90–4.54		
**Paid employment last 12 months (ref: No)**									
Yes	25.2	0.50**	**0.32**–**0.79**					0.79	0.45–1.40
**Age (ref: 18–24)**									
25–39	51.4	1.32	0.57–3.09	1.45	0.58–3.65	1.44	0.57–3.63	1.44	0.58–3.60
40–54	35.7	0.69	0.30–1.59	0.89	0.36–2.16	0.91	0.37–2.23	0.90	0.37–2.20
55plus	19.5	0.30**	**0.13**–**0.73**	0.40	0.16–1.04	0.40	1.54–1.05	0.45	0.17–1.12
**Gender (ref: Male)**									
Female	36.4	1.08	0.72–1.62	1.41	0.88–2.26	1.34	0.83–2.15	1.45	0.90–2.33
**Smoking status (ref: Smoker)**									
Long-term ex-smoker	19.1	0.31***	**0.19**–**0.51**	0.52*	**0.30**–**0.92**	0.48*	**0.27**–**0.86**	0.52*	**0.29**–**0.91**
Recent ex-smoker	47.6	1.19	0.49–2.90	1.46	0.54–3.9	1.51	0.56–4.04	1.50	0.56–4.05
**Vape status (ref: Daily)**									
Non-Daily	52.2	3.26***	**2.14**–**4.97**	2.88***	**1.71**–**4.83**	2.88***	**1.71**–**4.87**	2.87***	**1.71**–**4.83**
**SUTS-E (ref: Slight/Moderate)**									
No urges at all/Don’t know	38.8	1.22	0.65–2.30	1.22	0.59–2.56	1.27	0.60–2.67	1.21	0.58–2.52
Slight/Moderate (ref)	34.1	1		1		1		1	
Strong	35.1	1.04	0.61–1.78	0.89	0.48–1.63	0.90	0.49–1.67	1.07	0.55–2.08
Very/extremely strong	42.3	1.42	0.63–3.22	0.97	0.38–2.51	1.01	0.39–2.56	1.09	0.47–2.53
**MITSE**									
Don’t want to and intent to stop (ref)	41.5	1		1		1		1	
Want and intent to stop	58.5	2.58***	**1.71**–**3.90**	2.87***	**1.70**–**4.83**	3.07***	**1.88**–**5.00**	2.24***	**1.47**–**3.43**

*Notes.* Ref = reference group. OR = odds ratio. LCI = Lower confidence interval. UCI = Upper confidence interval. MITSE = Motivation and intention to stop using e-cigarettes within the next three months. SUTS-E = Strength of urges to use e-cigarettes. Significant associations (p < .05) are highlighted in bold. Multivariable Models were run with one socioeconomic indicator at a time. Regardless of the socioeconomic indicator used, the results did not change significantly. * *p* < .05, ** *p* < .01, *** *p* < .001.

## 4 Discussion

Overall, the findings of this study extend previous research suggesting a social gradient in e-cigarette use among long-term ex-smokers (Kock et al., 2019). We found evidence for an association between socioeconomic characteristics and motivation to stop using e-cigarettes, but only in the unadjusted model. Because this is an exploratory study we can only speculate as to why we found the particular pattern of results. For example, e-cigarettes users with higher income and education may be more likely to be aware that e-cigarettes contain the addictive substance nicotine and thus they may be more motivated to stop using e-cigarettes. However, previous studies (Brose, Brown, Hitchman, & McNeill, 2015) did not find evidence that harm perception differs by income or education. Further, lower income and education is associated with higher nicotine dependence (Chen, Machiorlatti, Krebs, & Muscat, 2019) which in turn may be associated with less motivation to stop using e-cigarettes. Lastly, it may be that e-cigarette users in paid employment perceive more stress and use e-cigarettes as a coping strategy. This may in turn have prevented them from both being motivated to stop and making an attempt to stop using e-cigarettes. Following our results, future research may focus on the underlying mechanisms of socioeconomic differences in the motivation to stop using e-cigarettes. Although our findings suggest that paid employment predicted attempts to stop using e-cigarettes in the unadjusted model, this association did not hold in the adjusted models. This finding is in line with smoking cessation literature suggesting that socioeconomic disadvantage is not strongly associated with quit attempts (Partos, Borland, & Siahpush, 2012). The differences in odds ratios in the adjusted and unadjusted models suggests that the association between socioeconomic characteristics and attempts to stop using e-cigarettes may be confounded by other variables such as smoking status or demographics. However, we found an association between smoking status and attempts to stop using e-cigarettes. It is possible that long-term ex-smokers have formed a habit of vaping which recent-ex-smokers have not yet established. The results also showed that motivation and intention to stop using e-cigarettes was associated with attempts to stop using e-cigarettes. This is in line with research on cigarette smoking which suggests that motivation to quit smoking predicts incidence of attempts to quit cigarette smoking (Kotz et al., 2013, Ussher et al., 2016). With the results of our study suggesting differences in motivation to stop using e-cigarettes and an association between motivation to stop using e-cigarettes and attempts to stop using e-cigarettes, future studies might focus on the mediating role of motivation to stop using e-cigarettes between indicators of SES and stopping the use of e-cigarettes.

The major strength of this longitudinal study is that it was the first to explicitly explore the associations between motivation to stop e-cigarette use and socioeconomic characteristics and the associations between socioeconomic and smoking characteristics at baseline and e-cigarette discontinuation at follow-up. To our knowledge this has not been examined previously. Although this study provides the first insights into the social patterning of the discontinuation of e-cigarettes among smokers and ex-smokers, the findings must be considered in the light of some limitations. Firstly, the recruitment method is likely to have led to selection bias as the sample for this study was recruited from a panel that consisted of individuals who were interested in participating in research surveys in exchange for vouchers or entering prize draws. This may not be representative of the wider population as it is likely that groups with very low socioeconomic characteristics, multiple addictions or mental health disorders were not included in this sample. Including those groups in our sample could have potentially accentuated the effects of socioeconomic characteristics on motivation and attempts to stop using e-cigarettes. Another limitation of this study was the high attrition rate and that respondents lost to follow-up differed substantially from those retained. As the respondents who were lost to follow-up were more highly educated, had more income, and were less likely to have had paid employment in the last 12 months, inclusion of those respondents who were lost to follow-up may have potentially weakened the results. Further, we used an adapted version of the Strengths of urges to smoke (SUTS) questionnaire to assess e-cigarette dependence and an adapted version of motivation to stop smoking to assess motivation to stop vaping. Both measures are yet to be validated for e-cigarette use. Finally, as with all surveys of this nature the data rely on self-report and thus may be subject to bias and error. By removing inconsistent data and running sensitivity analyses, we tried to reduce the impact of such observations. The findings of this study warrant replication with larger sample sizes and refined measures to better understand how socioeconomic characteristics are associated with the use and discontinuation of e-cigarettes, especially after a smoking cessation attempt, and how this may affect health inequalities. Future research may further consider including additional theoretically justified socioeconomic characteristics such as occupation or composite measures that include a variety of facets of socioeconomic status.

Following from the finding of this study, targeted public health campaigns among vapers with lower education/income may help increase the motivation to stop using e-cigarettes. In addition, workplace support may further help those who were in paid employment over the last 12 months in increasing their motivation to stop using e-cigarettes. However, it is important to highlight that this should be delivered with caution in order to avoid increasing the risk of relapse to smoking. In addition, further exploration into the habits surrounding vaping after successful smoking cessation is warranted.

## 5 Conclusion

Higher socio-economic status may be associated with higher motivation to stop using e-cigarettes but with lower likelihood of trying to do so. Respondents with higher education and higher income were more likely to be motivated to stop using e-cigarettes. Respondents with paid employment may be less likely to attempt to stop using e-cigarettes. However, when controlling for covariates we no longer found evidence for an association between socioeconomic characteristics and motivation to stop and attempts to stop using e-cigarettes. Further research on the social patterning of the discontinuation of e-cigarettes is needed and how this might affect health inequalities.

## Funding

10.13039/501100000289Cancer Research UK (C52999/A21496; C57277/A23884) funded the surveys reported in this manuscript. Cancer Research UK had no role in the study design, collection, analysis or interpretation of the data, writing the manuscript, or the decision to submit the paper for publication.

## CRediT authorship contribution statement

**Tina Jahnel:** Writing - original draft, Formal analysis, Conceptualization. **Stuart G. Ferguson:** Writing - review & editing, Supervision, Conceptualization. **Timea Partos:** Writing - review & editing. **Leonie S. Brose:** Writing - review & editing, Supervision, Funding acquisition, Conceptualization, Methodology.

## Declaration of Competing Interest

The authors declare that they have no known competing financial interests or personal relationships that could have appeared to influence the work reported in this paper.
