# Supplementary material for: Socioeconomic differences in the motivation to stop using e-cigarettes and attempts to do so
Source: Addict Behav Rep. 2020 Jan 12;11:100247. doi: 10.1016/j.abrep.2020.100247 (PMC7244924; doi:10.1016/j.abrep.2020.100247)
Supplement: Supplementary Data 1 [file mmc1.docx]

**Supplementary Material**

Box S1: Items used for analysis including original categorisation

| **Formal education**  What is the highest level of formal education that you have completed?   1. Primary or secondary school/vocational level 1 & 2/trade apprenticeship^0^ 2. Secondary school advanced/vocational level 3^0^ 3. Further education/ training college below degree level^0^ 4. Some university^1^ 5. Completed university degree^1^ 6. Post-graduate degree^1^ 7. Prefer not to say^0^ 8. Don’t know^0^   ^0^Categorised as low education; ^1^Categorised as high education |
| --- |
| **Income**  Which of the following categories best describes your ANNUAL household income, that is the total income before taxes, or gross income, of all persons in your household combined, for one year?   1. Under £6,500^1^ 2. £6,501-15,000^1^ 3. £15,001-30,000^1^ 4. £30,001-40,000^2^ 5. £40,001-50,000^2^ 6. £50,001-65,000^2^ 7. £65,001-95,000^2^ 8. £95,001 and over^2^ 9. Prefer not to say^0^ 10. Don’t know^0^   ^1^ Categorised as low income; ^2^ Categorised as high income; ^0^Don’t know/Prefer not to say |
| **Paid employment**  Apart from any unpaid work, have you worked at all in the last 12 months?   1. Yes - full or part time^1^ 2. Yes - whilst registered as a full-time student^1^ 3. No^0^ 4. Don't know^0^   ^1^Yes; ^0^No |
| **Smoking status** Could you please tell us which of the following best applies to you now?   1. I smoke cigarettes (including hand-rolled) every day^1^ 2. I smoke cigarettes (including hand-rolled), but not every day^1^ 3. I do not smoke cigarettes at all, but I do smoke tobacco of some kind (e.g. pipe or cigar)^1^ 4. I have stopped smoking completely in the last year (i.e. since May 2015)/since the last survey in December 2014) [In Wave 4, different time spans for previous participants and new recruits; in Wave 5, May/June 2016 for all participants] 5. I stopped smoking completely more than a year ago (i.e. before May 2015)/before the last survey in December 2014 [In Wave 4, different time spans for previous participants and new recruits; in Wave 5, May/June 2016 for all participants]^2^ 6. I have never been a smoker^^^   If [d]: How long ago did your most recent quit attempt start? By most recent, we  mean the last time you tried to quit smoking.   1. In the last week^3^ 2. More than a week and up to a month^3^ 3. More than 1 month and up to 2 months^3^ 4. More than 2 months and up to 3 months^3^ 5. More than 3 months and up to 6 months^2^ 6. More than 6 months and up to a year^2^ 7. More than a year and up to 15 months^2^ 8. Don’t know / can’t remember*   ^1^Categorised as current smoker; ^2^Long-term ex-smoker; ^3^Recent ex-smoker; ^*^Excluded; ^^^ Not included in survey |
| **Vaping status** Could you please tell us which of the following best applies to you now?   1. I currently vape/use e-cigarettes daily^1^ 2. I currently vape/use e-cigarettes but not every day^1^ 3. I have tried vaping/an e-cigarette once or a few times^^^ 4. I stopped vaping/using e-cigarettes since the in the last year [In wave 5, May/June 2016] ^^^ 5. I stopped vaping/using e-cigarettes over a year ago [In wave 5, May/June 2016] ^^^ 6. I have never vaped/used e-cigarettes. ^^^   ^1^Categorised as daily and non-daily e-cigarettes users; ^^^ Not included in sample |
| **Motivation and intention to stop using e-cigarettes**  Thinking about your electronic cigarette or vaping device use, which of the following best describes you?   1. I REALLY want to stop using e-cigarettes / vaping devices and intend to in the next month^1^ 2. I REALLY want to stop using e-cigarettes / vaping devices and intend to in the next 3 months^1^ 3. I want to stop using e-cigarettes / vaping devices and hope to soon^1^ 4. I REALLY want to stop using e-cigarettes / vaping devices but I don't know when I will^1^ 5. I want to stop using e-cigarettes / vaping devices but haven't thought about when^1^ 6. I think I should stop using e-cigarettes / vaping devices but don't really want to^0^ 7. I don't want to stop using e-cigarettes / vaping devices^0^ 8. Don't know^0^   ^1^ High motivation and intention to stop e-cigarettes; ^0^ Low motivation and intention to stop e-cigarettes |
| **Number of attempts to stop using e-cigarettes**  How many serious attempts to stop using an e-cigarette/ vaping device (if any) have you made in the last 12 months, that is since May 2015?  By serious attempt we mean you decided that you would try to make sure you never used an e-cigarette or vaping device again. Please include any attempt that you are currently making and please include any successful or unsuccessful attempt you made in this time.   1. None^0^ 2. One^1^ 3. Two^1^ 4. Three or more^1^ 5. Not sure, but at least one^1^   ^0^ No attempt; ^1^ At least one attempt |
| **Strength of urges to use e-cigarettes**  How much of the time have you felt the urge to use your e-cigarette/ vape in the past 24 hours?   1. Not at all^0^ 2. A little of the time 3. Some of the time 4. A lot of the time 5. Almost all of the time 6. All the time 7. Don’t know^0^   [If b-f]: In general, how strong have these urges been?   1. Slight^1^ 2. Moderate^1^ 3. Strong^2^ 4. Very Strong^3^ 5. Extremely Strong^3^ 6. Don't know^0^   ^0^No urges, Don’t know; ^1^ Slight/moderate; ^2^ Strong; ^3^ Very strong/Extremely strong |
| **Strength of urges to smoke** How much of the time have you felt the urge to smoke in the past 24 hours?   1. Not at all^0^ 2. A little of the time 3. Some of the time 4. A lot of the time 5. Almost all of the time 6. All the time 7. Don’t know   [If b-f]: In general, how strong have the urges to smoke been?   1. Slight^1^ 2. Moderate^1^ 3. Strong^2^ 4. Very Strong^3^ 5. Extremely Strong^3^ 6. Don't know^0^   ^0^No urges, Don’t know; ^1^ Slight/moderate; ^2^ Strong; ^3^ Very strong/Extremely strong |

Table S1: Respondent characteristics for those followed up at baseline (n = 1720) and comparison with those lost to follow-up (n = 1614)

|  |  |  | **Followed up**  **n = 1720** |  | **Lost to follow-up**  **n = 1614** |  | **Comparison** | |
| --- | --- | --- | --- | --- | --- | --- | --- | --- |
| **Education** |  |  |  |  |  |  | | **χ2= 7.4, *p* < .006** |
|  | Low |  | 1057 (61.5) |  | 917 (56.8) |  | |  |
|  | High |  | 663 (38.5) |  | 697 (43.2) |  | |  |
| **Annual Income** |  |  |  |  |  |  | | **χ2= 17.1, *p* < .001** |
|  | Under £6,501-30,000 |  | 886 (51.5) |  | 766 (47.5) |  | |  |
|  | £30,001 and over |  | 667 (38.8) |  | 731 (45.3) |  | |  |
|  | Don't know/Prefer not to say |  | 167 (9.7) |  | 117 (7.2) |  | |  |
| **Paid employment last 12 months** |  |  |  |  |  |  | | **χ2 = 12.2, *p <*.001** |
|  | Yes |  | 626 (36.4) |  | 495 (30.7) |  | |  |
|  | No |  | 1094 (63.6) |  | 1119 (69.3) |  | |  |
| **Age, mean (SD)** |  |  | 48.1 (15.1) |  | 41.1 (15.1) |  | | **t = 13.4, *p* < .001** |
| **Gender** |  |  |  |  |  |  | | χ2= 2.6, *p* =.108 |
|  | Female |  | 758 (44.1) |  | 756 (46.8) |  | |  |
|  | Male |  | 962 (55.9) |  | 858 (53.2) |  | |  |
| **Ethnic group** |  |  |  |  |  |  | |  |
|  | White |  | 1604 (93.3) |  | 1456 (90.8) |  | | **χ2= 7.2, *p* = .027** |
|  | Not White |  | 104 (6) |  | 131 (8.1) |  | |  |
|  | Don’t know/ Prefer not to say |  | 12 (0.7) |  | 18 (1.1) |  | |  |
| **Smoking status** |  |  |  |  |  |  | | χ2= 0.79, *p* = .674 |
|  | Smoker |  | 1247 (72.5) |  | 1182 (73.2) |  | |  |
|  | Long-term ex-smoker |  | 358 (20.8) |  | 314 (19.5) |  | |  |
|  | Recent ex-smoker |  | 58 (3.4) |  | 54 (3.3) |  | |  |
| **Vaping status** |  |  |  |  |  |  | | χ2= 0.89, *p* = .344 |
|  | Daily |  | 294 (17.1) |  | 316 (19.6) |  | |  |
|  | Non-daily |  | 212 (12.3) |  | 202 (12.5) |  | |  |
| **MITSE** |  |  |  |  |  |  | | χ2= 3.10, *p* = .079 |
|  | Want and intent to stop |  | 233 (13.5) |  | 267 (16.5) |  | |  |
|  | Don’t want/intent to stop |  | 273 (15.9) |  | 251 (15.6) |  | |  |
| **Strength of urges to use e-cigarettes** |  |  |  |  |  |  | | χ2= 4.09, *p* = .252 |
|  | No urges at all/Don’t know |  | 117 (6.8) |  | 118 (7.3) |  | |  |
|  | Slight/Moderate |  | 326 (19.0) |  | 317 (19.6) |  | |  |
|  | Strong |  | 102 (5.9) |  | 118 (7.3) |  | |  |
|  | Very strong/ Extremely strong |  | 31 (1.8) |  | 47 (2.9) |  | |  |
| **Strength of urges to smoke^a^** |  |  |  |  |  |  | | χ2= 2.804, *p* = .423 |
|  | No urges at all/ Don’t know |  | 237 (13.8) |  | 226 (14.0) |  | |  |
|  | Slight/ Moderate |  | 809 (47.0) |  | 724 (44.9) |  | |  |
|  | Strong |  | 307 (17.8) |  | 320 (19.8) |  | |  |
|  | Very strong/ Extremely Strong |  | 155 (9.0) |  | 138 (8.6) |  | |  |

Notes. Entries are N (%) unless otherwise stated. Significant associations (p < .05) are highlighted in bold. MITSE=Motivation and intention to stop using e-cigarettes within the next three months. ^a^Ex-smokers who stopped more than 1 year ago (n=178) were not asked for urges to smoke.
